# The Phytochemical and Functional Characterization of the Aerial Parts of *Artemisa alba Turra* (*Asteraceae*) Grown in Romania

**DOI:** 10.3390/foods14081389

**Published:** 2025-04-17

**Authors:** Mădălina Țicolea, Raluca Maria Pop, Marcel Pârvu, Lia-Oxana Usatiuc, Ana Uifălean, Valeria Alvarez Brito, Eva Fischer-Fodor, Floricuța Ranga, Crina Claudia Rusu, Maria Crisan, Bianca Bosca, Florinela Adriana Cătoi, Alina Elena Pârvu

**Affiliations:** 1 Department of Morpho-Functional Sciences, Discipline of Pathophysiology, “Iuliu Haţieganu” University of Medicine and Pharmacy, 400012 Cluj-Napoca, Romania; madalina.ticolea@umfcluj.ro (M.Ț.); lia.usatiuc@umfcluj.ro (L.-O.U.); adriana.catoi@umfcluj.ro (F.A.C.); parvualinaelena@umfcluj.ro (A.E.P.); 2 Department of Morpho-Functional Sciences, Discipline of Pharmacology, Toxicology and Clinical Pharmacology, “Iuliu Haţieganu” University of Medicine and Pharmacy, 400337 Cluj-Napoca, Romania; 3 Department of Biology, Babes-Bolyai University, 400015 Cluj-Napoca, Romania; marcel.parvu@ubbcluj.ro; 4 Faculty of Medicine, Autonomous University of Nuevo Leon, Monterrey 64460, Mexico; valeria.alvarez.brito@hotmail.com; 5 Tumor Biology Department, The Oncology Institute “I. Chiricuță”, 400015 Cluj-Napoca, Romania; fischer.eva@iocn.ro; 6 Food Science and Technology, Department of Food Science, University of Agricultural Science and Veterinary Medicine Cluj-Napoca, 400372 Cluj-Napoca, Romania; 7 Department of Nephrology, “Iuliu Hatieganu” University of Medicine and Pharmacy, 400012 Cluj-Napoca, Romania; claudia.rusu@umfcluj.ro; 8 “Mihai Manasia” Nephrology and Dialysis Clinic, County Emergency Clinical Hospital Cluj, 400347 Cluj-Napoca, Romania; 9 Department of Morpho-Functional Sciences, Discipline of Histology, “Iuliu Haţieganu” University of Medicine and Pharmacy, 400337 Cluj-Napoca, Romania; maria.crisan@umfcluj.ro (M.C.); bianca.bosca@umfcluj.ro (B.B.)

**Keywords:** *Artemisia alba Turra*, phytochemistry, inflammation, oxidative stress, antiproliferative

## Abstract

*Artemisia alba Turra* is a plant used in folk medicine. Due to its significant polymorphism, there are different chemotypes. This study aimed to characterize the specific chemotypes and evaluate the anti-inflammatory, antioxidant, and antiproliferative potential of an ethanol extract of *A. alba Turra* aerial parts prepared from plants harvested from the “Alexandru Borza” Botanical Garden, Cluj-Napoca, Romania. The extract phytochemical analysis performed by measuring total polyphenol content (3.4 ± 0.21 mgGAE/g d.w.), total polyphenolic flavonoids (147.12 ± 10.09 mg QE/100 g d.w.), and HPLC-ESI MS polyphenol profiles indicated that in the *A. alba Tura* extract from the hydroxycinnamic acids chlorogenic acid, caffeoyl tartaric acid, 3,4-dicaffeoylquinic acid, 3,5-dicaffeoylquinic acid, and 4,5-dicaffeoylquinic acid had and from the flavonols, isorhamnetin-rutinoside and rutin had the highest concentration. The extract exhibited good in vitro and in vivo antioxidant activity by reducing oxidants without significant effects on antioxidants. The anti-inflammatory effect tested on rat turpentine oil-induced inflammation was indicated by the reduction in NLRP3 inflammasome markers, NfkB-p65, IL-1β, IL-18, caspase-1, and gasdermin D. The extract had in vitro antiproliferative activity against ovarian tumor cell lines at concentrations from 12.5 to 50 μg/mL, and this mechanism was linked to MDR and NF-κB modulation. *A. alba Turra* had no liver toxicity and reduced kidney injury associated with inflammation. These findings indicated that this specific Romanian chemotype of *A. alba Turra* has antioxidant, anti-inflammatory, and antiproliferative properties with potential applications as tumor microenvironment-targeted therapy.

## 1. Introduction

Cancer is the main cause of death worldwide. The tumor microenvironment (TME) is a highly structured ecosystem, consisting of tumor cells, endothelial cells, fibroblasts, extracellular matrix, immune cells, and secreted cytokines, which contributes in various degrees to cell proliferation, invasion and metastasis, resistance to cell death, angiogenesis, tumor-promoting inflammation, and the avoidance of immune destruction [1]. Accordingly, today, anticancer treatment has changed, and to antineoplastic treatments, such as surgery, chemotherapy, radiotherapy, or immunotherapy, TME-targeted therapies have been added [2]. The idea of “normalization” is to revert the tumor-favoring TME to a normal tissue microenvironment, which reduces early tumor development and improves the anticancer therapy effect [1].

In TME, the intercellular dialog is regulated through cell–cell contact mediated by adhesion molecules and paracrine signaling through the secretion of cytokines, chemokines, growth factors, and proteases. Cancer-induced inflammation is characterized by an abnormal adaptive innate immune cell response that leads to chronic and damaging inflammation, like wounds that do not heal [3]. In physiological conditions, antioxidants reduce ROS formation and prevent associated cellular injury. In oxidative stress disease conditions, the ROS/antioxidant balance is disturbed because of an antioxidant deficiency or excessive cellular ROS production. Moreover, the excess formation of ROS results in the oxidation of cellular macromolecules like lipids, proteins, and deoxyribose nucleic acids (DNA) [4]. Chronically activated TME-associated macrophages and neutrophils contribute directly to the oncogenic process via the production of reactive oxygen species (ROS) and reactive nitrogen species (RNS) that can directly induce DNA damage in epithelial cells through oxidative and nitrosative stress [4].

Thus, in cancer, an actual therapeutic option is to target both inflammation and oxidative stress as part of the TME-targeted therapies [5].

The use of natural compounds and plant extracts that have a high antioxidant capacity has received much attention over the last few decades as TME-targeted therapies [6,7]. Plants contain metabolites, such as phenolic compounds, flavonoids, and alkaloids, with a high range of biological activity, like anti-inflammatory, antioxidant, antiproliferative, and chemoprotective effects [8]. Plant antioxidants can balance the ROS/antioxidant ratio and reduce or eliminate cellular oxidative stress. One of the advantages of these polymolecular therapies is their ability to interfere with different targets in the tumor [7]. Moreover, these natural molecules have minimal side effects and low cost [9].

Plants from the genus *Artemisia* belong to the *Asteraceae* family and are important in folk medicine. The over 500 varieties of *Artemisia* are spread over temperate areas of Asia, Europe, and North America [10,11,12]. The phytochemical composition of *A. herba-alba* is complex and demonstrates a significant polymorphism that allowed the identification of different chemotypes worldwide [8,13]. *A. herba-alba* also contains the antimalarial drug artemisinin in amounts that are possibly greater than in *A. annua.* As a consequence, in folk medicine, *A. herba-alba* has been utilized differently in antimalarial, antiviral, antitumor, antipyretic, antihemorrhagic, anticoagulant, antianginal, antioxidant, antihepatitis, and antispasmodic applications [8,10,14,15].

The current study’s first aim was to characterize the specific chemotype of an *A. alba Turra* ethanol extract prepared from plants harvested from the Alexandru Borza Botanical Garden, Cluj-Napoca, Romania. The second aim was to evaluate the anti-inflammatory, antioxidant, and antiproliferative potential of this *A. alba Turra* ethanol extract.

## 2. Materials and Methods

### 2.1. Chemicals

Acetonitrile, ethanol, methanol, diethylether, ammonium iron (II) sulfate ((NH_4−_)_2_Fe(SO_4−_)_2_·6H_2_O), vanadium (III) chloride (VCl_3_), sulfanilamide (C_6_H_8_N_2_O_2_S), N-(1-Naphthyl) ethylenediamine dihydrochloride (C_12_H_14_N_2_), acetic acid, sulfuric acid, acetic acid, hydrochloric acid, xylenol orange [o-cresosulfonphthalein-3,3-bis(sodiummethyliminodiacetate)], hydrochloric acid, glycerol, hydrogen peroxide (H_2_O_2_), ortho-dianisidine dihydrochloride (3-3′-dimethoxybenzidine), sodium nitrite, thiobarbituric acid, o-Phthalaldehydealuminum chloride-1-Ethyl-3-methylimidazolium chloride, sodium hydroxide, sodium nitroprusside, sodium carbonate, trichloroacetic acid, chloramine-T, thiobarbituric acid, potassium iodide, Folin–Ciocalteu′s phenol reagent and 5,5′-Dithio-bis-(2-nitrobenzoic acid) were purchased from Merck (Darmstadt, Germany); rutin, gallic acid, chlorogenic acid, luteolin, quercetin analytical standards were bought from Sigma (St. Louis, MO, USA); trolox (6-hydroxy-2,5,7,8-tetramethylchroman-2-carboxylic acid) was sourced from Alfa-Aesar (Karlsruhe, Germany); rat’s ELISA kits were supplied by Elabscience Bionovation Inc. (Houston, TX, USA) and My Biosource (San Diego, CA, USA); and reagents for ALT, AST, urea and creatinine were purchased from Bio Systems Diagnostic (Popesti-Leordeni, Ilfov, Romania).

All chemicals utilized in this study were of ultrapure grade, ensuring high purity standards. Type I reagent-grade deionized water was consistently employed throughout the research. For HPLC analysis, ultrapure water was produced using the Direct-Q UV system from Millipore (Burlington, MA, USA), ensuring optimal analytical performance.

### 2.2. Plant Collection and Extraction Protocol

*A. alba Turra* (fresh stems and leaves) was obtained in June 2021 from the Botanical Garden “Alexandru Borza” in Cluj-Napoca (46°45′36″ N and 23°35′13″ E). The plant was taxonomically identified and extracted using the cold repercolation method [16]. Shortly, three consecutive applications of identical menstruum were repercolated to the leaves and stems. In every percolator, the plant material was cut into 1–1.5 cm fragments and loaded in the first (150 g), in the second (90 g), and in the third percolator (60 g). The plant material was soaked with 150 mL 30% ethanol, macerated (for two days), and finally percolated. The percolated fractions (60 mL, 90 mL, and 120 mL, respectively) from the first, the second, and the third percolator were collected and mixed. Finally, the extract had a ratio of 1 g of fresh plant to 1.2 mL of extract (*w*:*v*) in 30% ethanol.

### 2.3. Phytochemical Analysis

Total Polyphenol Content (TPC): TPC was measured using the Folin–Ciocâlteu method. First, the extract (2 mL) was diluted (25 times) and mixed with Folin–Ciocâlteu reagent (1 mL) and distilled water (10.0 mL). Then, the mixture was diluted (up to 25 mL) by adding sodium carbonate solution (290 g/L). Afterward, the mixture was incubated for 30 min in darkness conditions, and the absorbance was read at 760 nm. The TPC values were expressed as gallic acid equivalents (GAE) (R^2^ = 0.999), mg GAE/g d.w. herbal material [17].

Total Flavonoid Content (TFC): TFC was determined using a colorimetric method. Briefly, the extract (5 mL) was mixed with sodium acetate (5 mL of 100 g/L) and aluminum chloride (3.0 mL of 25 g/L). Then, the mixture was diluted (up to 25 mL) with methanol. The absorbance was read at 430 nm. The TFC was calculated as quercetin equivalents (R^2^ = 0.999), mg QE/g d.w. herbal material [17].

TPC and TFC were measured with a JASCO V-530 UV–vis spectrophotometer (Jasco International Co., Ltd., Tokyo, Japan), and the assays were performed in triplicate.

Phenolic compounds in *A. alba Turra* ethanol extract were separated using an Agilent 1200 HPLC equipped with a DAD detector (diode array detection) coupled with an Agilent 6110 MS (Santa Clara, CA, USA) single quadrupole mass spectrometer. The phenolic compounds were separated on an XDB C18 Eclipse column at room temperature, employing a gradient elution with mobile phases containing acetic acid and acetonitrile. The elution gradient started with 5% mobile phase B (0.1% acetic acid in distilled water, *v*/*v*) and increased to 40% in two minutes. Afterward, the percentage of B was increased to 90 in 16 min and maintained for 2 min. After 20 min, the percentage was decreased in 5 min to the initial conditions (5% B) and maintained for another 5 min. The mobile phase A was acetonitrile. The flow rate was set at 0.5 mL/min, the injection volume at 10 uL, and the detection wavelengths at 280 nm (specific to phenolic acids) and 340 nm (specific to flavonoids). Prior injection, samples were filtered using 0.45 um membrane filters (Fisher Scientific, AMEX, Bucharest, Romania). Mass spectrometric analysis utilized positive ion mode in the *m*/*z* range of 100–1000. Phenolic compounds were identified based on UV–visible spectra, retention times, mass spectra, co-chromatography with standards, the literature data, and the Phenol-Explorer database. Calibration curves were generated using standard solutions, with gallic acid (R^2^ = 0.9978), LOD = 0.35 μg/mL, LOQ = 1.05 μg/mL, chlorogenic acid (R^2^ = 0.9937), LOD = 0.41 μg/mL, LOQ = 1.64 μg/mL, luteolin (R^2^ = 0.9972), LOD = 0.26 μg/mL, LOQ = 0.95 μg/mL, and rutin (R^2^ = 0.9981), LOD = 0.21 μg/mL, LOQ = 0.84 μg/mL used for quantification [17,18,19].

### 2.4. In Vitro Antioxidant Activity Analysis

For DPPH radical scavenging capacity measurement, 2 mL of extract was added to the DPPH methanol solution (2 mL of 0.1 g/L). After 30 min of incubation (at 37 °C; dark conditions), the absorbance was read (at 517 nm) [17]. Then, the percentage of radical scavenging activity (AA%) was calculated using the following formula: AA% = [(A control − A sample)/A control] × 100. AA% was converted to μg Trolox equiv./mL using a Trolox standard solution calibration curve (0.5–5 μg/mL, R^2^ = 0.997). For a good antioxidant capacity, IC50 ≤ 50 µg TE/mL; for a moderate antioxidant capacity, IC50 is 50–100 µgTE/mL; and for a negligible antioxidant capacity, IC50 is ≥200 µg TE/mL [20].

The ferric reducing antioxidant power (FRAP) assay measures the reducing power of an extract. Briefly, FRAP reagent (3.4 μL) was mixed with the extract (100 μL), and after 30 min, absorbance was read at 593 nm. The results were reported as IC50 values in mg mgTE/mL [17,20].

The hydrogen peroxide (H_2_O_2_) radical scavenging assay measured the ability of the extract to neutralize H_2_O_2_. Briefly, the extract (0.1 mL) was diluted with phosphate buffer (50 mM; pH 7.4) to 0.4 mL, and then H_2_O_2_ solution (0.6 mL of 2 mM) was added. The mixture was vortexed (for 10 min). The absorbance was measured at 230 nm. The hydrogen peroxide percentage scavenging activity was calculated with the following formula: H_2_O_2_ radical scavenging % = (A control − A sample/A control) × 100. The results are expressed as IC50 in µgTE/mL plant extract.

The nitric oxide (NO) radical scavenging assay measures the ability of the extract to scavenge radicals. Briefly, the extract (0.5 mL) was added to a mixture of sodium nitroprusside (2 mL) and phosphate-buffered saline (0.5 mL of pH 7.4). After incubation (2.5 h at 25 °C), the reaction mixture (0.5 mL) was added to sulphanilic acid (1 mL of 0.33% in 20% glacial acetic acid), and after 5 min, naphthylethylene–diamine–dihydrochloride (1 mL of 0.1% *w*/*v*) was added. The final solution was vortexed and incubated for 30 min, absorbance was read at 546 nm, and the percentage inhibition was calculated with the following formula: NO radical scavenging % = (A blank − A sample/A blank) × 100. The results are expressed IC50 in µg quercetin equivalents per mL extract [20].

The in vitro antioxidant assays were measured using a JASCO V-530 UV–vis spectrophotometer (Jasco International Co., Ltd., Tokyo, Japan), and the assays were performed in triplicate.

### 2.5. Antiproliferative Activity

This study also assessed the antiproliferative effects of *A. alba Turra* on human A2780 (ovarian adenocarcinoma), OVCAR-3 (high-grade ovarian serous adenocarcinoma), OAW-42 (ovarian cystadenocarcinoma), and HaCaT (normal keratinocyte) with the MTT cell growth inhibition assay. Briefly, tumor and normal cells seeded on 96-well flat-bottom microplates at a concentration of 2 × 10^4^ cells/well in 190 μL appropriate cell culture medium were treated by adding 10 μL extract (6 serial concentrations from 1.56 to 50 μg/mL) in each well and 10 μL of PBS to reference cells. After 72 h, the cell culture medium was withdrawn, and 100 µL of 1 mg/mL MTT solution (from Merck KGaA via Sigma-Aldrich, St. Louis, MO, USA) was added to each well and incubated at 37 °C. After 1 h of incubation, the MTT solution was removed and replaced with 150 µL dimethyl sulfoxide (from Titolchimica, Pontecchio Polesine, Province of Rovigo, Veneto region, Italy), and absorbance was immediately measured at 570 nm with a BioTek Synergy2 multiplate reader, and the IC50 was calculated from the dose–response curves generated by Graph Pad Prism 5 (GraphPad Software, Inc., La Jolla, CA, USA) [21].

A fluorimetric multidrug resistance protein (MDR) assay kit was used to evaluate the potential modulation of the *A. alba Turra* extract on tumor cell lines’ resistance to chemotherapy. The cells were treated with the most relevant concentrations related to the cell growth inhibition of *A. alba Turra* extracts, 25.00, 12.50, 6.25, and 3.13 μg/mL, and one of the wells was left without treatment. The fluorescence intensity was detected at excitation (490 nm) and emission (525 nm) wavelengths [22].

The NF-κB p65 protein in the cell lines was measured semi-quantitatively by an ELISA kit, the standard being provided by the kit. The same concentrations were used as in MDR testing [23].

### 2.6. In Vivo Experimental Design

#### 2.6.1. Animal Subjects

Adult male albino Wistar rats (200–250 g) were sourced from the “Iuliu Hațieganu” University of Medicine and Pharmacy in Cluj-Napoca, Romania. They were housed under controlled conditions with access to a standard diet and water. All procedures followed Directive 2010/63/EU and Romanian Law 43/2014, ensuring animal welfare. The project received approval from the Veterinary Sanitary Direction and Food Safety in Cluj-Napoca (No. 303/04.04.2022) and was conducted in triplicate.

#### 2.6.2. Experimental Protocol

**Figure 1:**
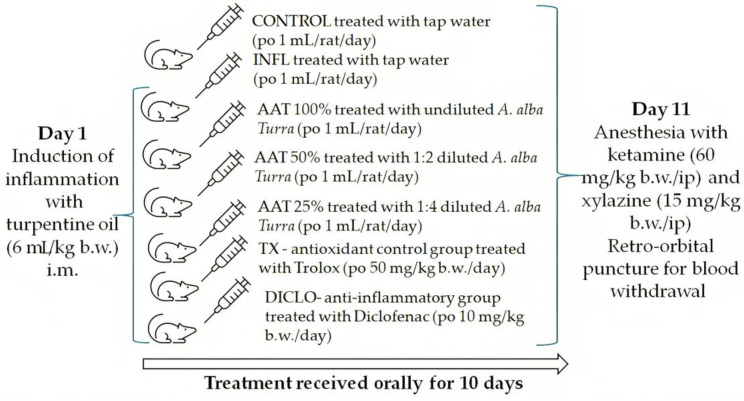
Experimental protocol. Adult male Wistar rats were put into seven groups (n = 9): negative CONTROL; six groups with turpentine oil-induced inflammation treated by gavage for 10 days as follows: INFL with no treatment, AAT 100% treated with undiluted *Artemisia alba Turra* extract, AAT 50% treated with 1:2 diluted *Artemisia alba Turra* extract, AAT 25% treated with 1:4 diluted *Artemisia alba Turra* extract, TX treated with TROLOX, DICLO treated with diclofenac. On the 11th day, animals were anesthetized and blood was drawn for further analysis. INFL—inflammation; AAT—*Artemisia alba Turra*; TX—trolox; DICLO—diclofenac.

The adult male Wistar rats (200–250 g b.w.) were randomly assigned to 7 groups (n = 9). On day 1, inflammation was induced with a turpentine oil intramuscular (i.m.) injection, except in the negative control group (CONTROL). From day 1, the animals were treated by gavage for 10 days as follows: the CONTROL and inflammation group (INFL) received tap water, the *A. alba Turra* (AAT) experimental groups received different dilutions of the plant extract in distilled water (100%, 50%, and 25%), the antioxidant control group was given Trolox and the anti-inflammatory drug group received diclofenac (DICLO). On day 11, the animals were anesthetized, blood was drawn, and serum was stored for later use. The protocol is detailed in Figure 1.

#### 2.6.3. Oxidative Stress Analysis

Oxidative stress analysis concerned several serum biomarkers related to the oxidative stress processes as previously described.

The total oxidative status (TOS) was quantified using a colorimetric assay. In the presence of an oxidant in an acidic medium, ferrous ion (Fe^2+^) is oxidated to ferric ion (Fe^3+^), and the reaction with xylenol orange detects the presence of the ferric ion. The results are expressed as μM H_2_O_2_ Equiv./L [17,24].

The total antioxidant capacity (TAC) was measured using a colorimetric method. First, a standard Fe^2+^-o-dianisidyl solution underwent the Fenton reaction with a standard H_2_O_2_ solution, resulting in hydroxyl ⋅OH radicals. In the presence of an acid, ⋅OH oxidizes o-dianisidines to dianisidyl radicals. The antioxidants from the serum sample inhibit the oxidation reactions and the appearance of coloration [17,25]. The results are expressed as mmol Trolox equivalent per liter (mmol TE/L).

The oxidative stress index (OSI) is calculated using the following formula: OSI (Arbitrary Unit) = TOS (mM H_2_O_2_ Equiv./L)/TAR (mM TE/L) [17,26].

The DNA damage was evaluated through 8-hydroxydeoxyguanosine (8-OHdG) using an ELISA kit (E-EL-0028), following the manufacturer’s protocol and expressed in ng/mL [17,27].

Advanced oxidation protein products (AOPPs) were measured following the method developed by Witko-Sarsat et al. Briefly, for the sample of 200 mL of serum diluted 1/5 in PBS, 20 mL of acetic acid is added, and for the standard sample, 10 mL of 1.16 M potassium iodide (Sigma) is added to 200 mL of chloramine-T solution (0–100 mmol/L), followed by 20 mL of acetic acid. The absorbance of the mixture is immediately read at 340 nm against a blank containing 200 mL of PBS, 10 mL of potassium iodide, and 20 mL of acetic acid. Sample absorbance is read at 340 nm, and AOPP concentration is expressed as µM chloramine-T Equiv./L [17,28].

Malondialdehyde (MDA), an indicator of lipid peroxidation, was measured using the thiobarbituric acid (TBA) method. Accordingly, 0.1 mL of serum was mixed with 40% trichloroacetic acid (0.1 mL), followed by the addition of 0.67% TBA (0.2 mL). The mixture was heated in a boiling water bath (30 min) and then rapidly cooled in an ice bath. After centrifugation (3461× *g*, 5 min), the absorbance of the supernatant was recorded at 532 nm. Using an MDA standard curve, serum MDA concentration was expressed in nmol/mL [17,29].

The serum concentration of nitric oxide (NO) is assessed using the stable end products nitrites and nitrates. First, proteins are removed by adding a 3:1 (*v*/*v*) solution of methanol/diethyl ether [30] and nitrates are reduced to nitrites by adding 100 μL of 8 mg/mL vanadium (III) chloride to 100 μL of serum. Then, 100 μL Griess reagents (50 μL of SULF 2% and 50 μL of NEDD 0.1%) is added, incubation takes place at 37 °C for 30 min, the sample absorbance is read at 540 nm. Using a standard sodium nitrite curve, serum NOx is expressed as nitrite μmol/L [17,31,32].

The peroxynitrite formation was measured by evaluating 3-nitrotyrosine (3NT) using an ELISA kit (E-EL-0040) according to the manufacturer’s instructions, and the results were expressed as ng/mL [17,33]. The ELISA equipment was composed of an 800 TS ELISA microplate reader (Agilent Technologies Inc., Santa Clara, CA, USA) and a Biotek Microplate 50 TS plate washer (Agilent Technologies Inc., Santa Clara, CA, USA).

Total thiols (SH) are measured using the modified Ellman’s reagent. Briefly, 0.6 mL of 20 mM Tris-HCl buffer (pH 8.2) is added to 0.2 mL of the serum sample. Then, 0.04 mL of 10 mM DTNB in absolute methanol and 3.16 mL of absolute methanol are added. After incubation at room temperature for 15 min, supernatant absorbance is measured at 412 nm. Using a standard curve of glutathione (GSH) (0.25 to 2 mM), serum SH concentration is expressed as mM GSH/mL [17,26].

#### 2.6.4. Inflammatory Markers

We apply inflammation serum markers nuclear factor Kappa B p65 (NfκB-p65) (E-EL-RO674), interleukin 1 beta (IL-1β) (E-EL-0012), interleukin 18 (IL-18) (E-EL-R0567), and caspase-1(MBS265585) and gasdermin D (GSDMD) (MBS2705517) using ELISA kits according to the manufacturer’s guidelines. The results for NfκB-p65 and GSDMD were expressed as ng/mL, while IL-1β and IL-18 were expressed as pg/mL. The ELISA equipment used is described in the previous subsection.

#### 2.6.5. Toxicity Assessment

Liver toxicity was assessed by measuring serum alanine transaminase (ALT) and aspartate transaminase (AST), while renal toxicity was assessed by measuring serum urea and creatinine.

The measurement of oxidative stress, anti-inflammatory, and toxicity markers were performed spectrophotometrically (Jasco V-350, Jasco International Co., Ltd., Tokyo, Japan) using the appropriate ELISA techniques (the Biotek Microplate 50 TS washer coupled with the 800 TS ELISA microplate reader—Agilent Technologies Inc., Santa Clara, CA, USA) according to the manufacturer’s protocols.

### 2.7. Statistical Analysis

The results were presented as mean ± standard deviation (SD) for normally distributed data. Groups were compared using one-way analysis of variance (ANOVA) followed by the Bonferroni–Holm post hoc test. The Pearson test and principal component analysis (PCA) were applied for correlation analysis. A *p*-value < 0.05 was considered statistically significant. Statistical analyses were conducted using SPSS Statistics Version 26.0 for Windows (SPSS, Chicago, IL, USA) and GraphPad Prism Version 8.0 (GraphPad Software, San Diego, CA, USA).

## 3. Results

### 3.1. Phytochemical Analysis

The *A. alba Turra* ethanol extract TPC was 3.4 ± 0.21 mg GAE/g d.w. plant material and TFC was 147.12 ± 10.09 mg QE/100 g d.w. plant material.

**Figure 2:**
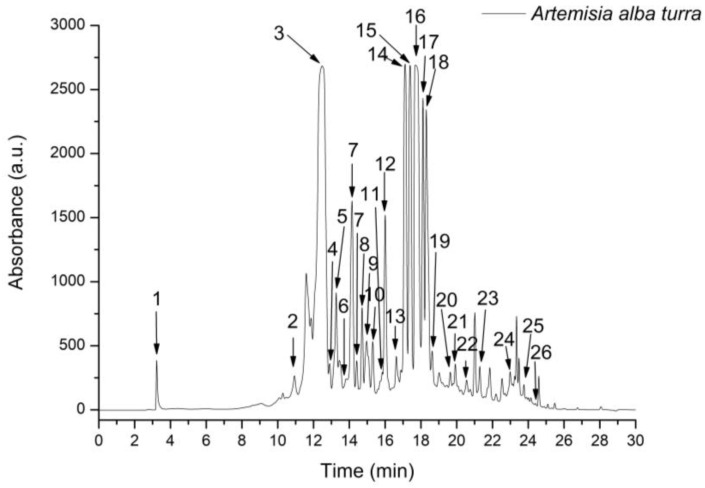
An HPLC-MS fingerprint of phenolic compounds extracted in *Artemisia alba Turra* ethanol extract. The peak identification is detailed in Table 1.

HPLC-ESI MS analysis showed that *A. alba Turra* ethanol extract had a rich content of phenolic compounds. A total of 26 compounds, including 20 phenolic acids and 6 flavonoids belonging to the flavone and flavonol classes, were evaluated. From the hydroxycinnamic acid subclass, chlorogenic acid, caffeoyl tartaric acid, 3,4-dicaffeoylquinic acid, 3,5-dicaffeoylquinic acid, and 4,5-dicaffeoylquinic acid had the highest concentration. From the flavonol subclass, isorhamnetin-rutinoside and rutin had the most significant values (Figure 2; Table 1).

### 3.2. In Vitro Antioxidant Activity

DPPH, FRAP, H_2_O_2_, and NO assays were used to estimate the in vitro antioxidant capacity of the *A. alba Turra* extract. The results obtained show that the *A. alba Turra* sample possesses moderate in vitro antioxidant activity. *A. alba Turra* extract DPPH, H_2_O_2,_ and NO scavenging capacities were smaller than those of Trolox (*p* < 0.001), and the FRAP assay result was smaller than that of quercetin (*p* < 0.001) (Table 2). Phenolic content correlates with the in vitro antioxidant activity of the extract (r^2^ = 0.72–0.91).

### 3.3. Antiproliferative Activity

**Figure 3:**
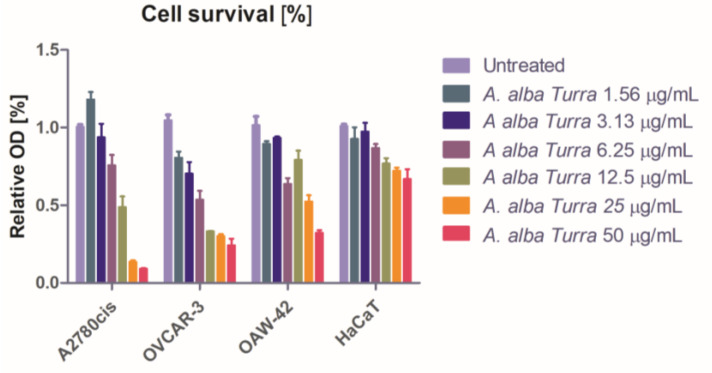
The compounds’ antiproliferative activity was expressed as a percent of the viable cells relative to the untreated control in A2780*cis*, OVCAR-3, OAW-42, and HaCaT cell populations subjected to *Artemisia alba Turra* extract at concentrations from 1.56 to 50 μg/mL.

The capacity of the *A. alba Turra* extract to inhibit tumor and normal cell growth was dose-dependent in the 72 h interval, with significant variation in the cell survival between doses and cell lines (*p* < 0.0001). There was a significant drop in cell survival in all cell lines treated with concentrations from 12.5 to 50 μg/mL. The *A. alba Turra* concentrations between 6.25 and 1.56 have had a notable inhibitory effect on A2780*cis* and OVCAR-3 cell lines (Figure 3).

For the higher concentrations, a decrease in proteins secreted in the growth medium (Figure S1) was observed. *A. alba Turra* concentration influenced the NF-κB secretion extremely significantly (*p* < 0.001), and the interaction of the extracts showed differences between the cell lines as well (*p* = 0.0069). In A2780*cis* and OVCAR-3 tumor cell lines, all concentrations gave an inhibitory effect in OAW-42, and only 25 and 12.5 μg/mL provided inhibition, while in the HaCaT cell line, the variation was inconsistent (Figure S2).

### 3.4. In Vivo Antioxidant Activity

The *A. alba Turra* extract significantly reduced TOS, OSI, and AOPP (*p* < 0.001). TX had a better inhibitory effect on TOS than *A. alba Turra* extract (*p* < 0.01). Also, it was observed that all dilutions induced a reduced decrease in MDA (*p* < 0.05). Only AAT 100% and AAT 50 lowered NO synthesis (*p* < 0.05), and 3NT was reduced only by AAT 100%. DNA oxidation measured through 8-OhdG was inhibited by *A. alba Turra* extract in a dose-dependent way, with AAT 100% having the best effect (*p* < 0.01). DICLO had a better inhibitory effect on NO, 3NT, and 8-OhdG than *A. alba Turra* extract (*p* < 0.01). None had an important effect on TAC (*p* > 0.05), but SH was significantly increased (*p* < 0.01) (Table 3).

### 3.5. In Vivo Anti-Inflammatory Activity

Inflammation increased significantly serum NfkB-p65, IL-1b, IL-18, caspase-1, and GSDMD (*p* < 0.001). The treatment with *A. alba Turra* extract caused an important reduction in serum NfkB-p65, IL-1b, and caspase-1 at all tested dilutions (*p* < 0.001). The inhibitory effect of *A. alba Turra* extract on IL-18 was not as significant (*p* < 0.01), and on GSDMD, the inhibitory activity was dose-dependent, with AAT 100% having the best effect (*p* < 0.001) (Table 4).

### 3.6. Liver and Renal Toxicity Assessment

Liver injury tests showed that AST and ALT were in normal ranges in INFL and all treated groups (*p* > 0.05). Renal dysfunction tests showed that creatinine and urea were increased after inflammation induction (*p* < 0.05). Only TX, DICLO, and AAT treatment lowered serum creatinine and urea (*p* < 0.01) (Table 5).

### 3.7. Correlation Analysis

**Figure 4:**
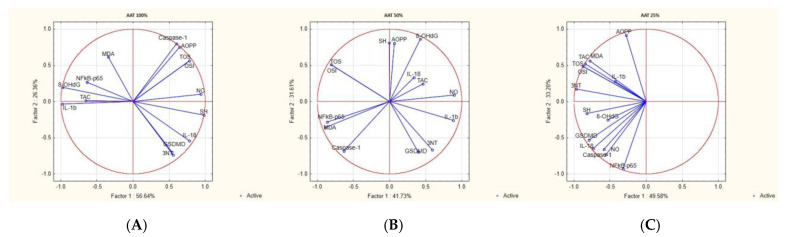
The PCA results of oxidative stress and inflammatory biomarkers based on the correlation matrix with PC1 and PC2 for *Artemisia alba Turra*: (**A**) PCA for *Artemisia alba Turra* 100% (AAT 100%); (**B**) PCA for *Artemisia alba Turra* 50% (AAT 50%); (**C**) PCA for *Artemisia alba Turra* 25% (AAT 25%). TOS—total oxidative status; TAC—total antioxidant capacity; OSI—oxidative stress index; AOPP—advanced oxidation protein product; MDA—malonyldialdehide; NO—nitrites and nitrates; 3NT—3-nitrotyrosine; 8-OHdG—8-hydroxydeoxyguanosine; SH—total thiols; NfkB-p65—Nuclear factor- κB; IL-1b—Interleukine 1-b; IL-18—Interleukine 18; GSDMD—Gasdermine D.

The treatments with ethanol extract had an inhibitory activity on inflammation and OS markers that varied according to the plant extract concentration. Parameter variability according to the comparisons of the first principal component (PC1) and the second component (PC2) was revealed by the PCA (Figure 4).

In AAT 100%, the inflammatory markers IL-18, caspase-1, and GSDMD were positively correlated with the OS markers TOS, OSI, AOPP, and NO. NF-kB-p65 and IL-1 were correlated with MDA and 8-OhdG. In AAT 50% inflammatory tests, NF-kB-p65, IL-1b, IL-18, caspase-1, and GSDMD were positively correlated with NO, 3NT, and MDA. TOS and OSI were correlated with AOPP and 8-OhdG. In AAT, 25% of the inflammatory markers IL-1b, IL-18, caspase-1, and GSDMD were positively correlated with TOS, OSI, MDA, 3NT, and 8-OhdG. In all treatment groups, inflammatory and OS markers correlated with creatinine (Figure 4).

## 4. Discussion

The present study characterized the specific chemotype of the ethanol extract from the aerial parts of *A. alba Turra* harvested from the Alexandru Borza Botanical Garden, Cluj-Napoca, Romania, and demonstrated that it has important associated anti-inflammatory, antioxidant, and antiproliferative activities.

Among the higher plants, *Artemisia* is considered one of the most evolved genera. A chemical analysis of secondary metabolites is always needed because *Artemisia* plants exhibit a great morphological polymorphism and diversity of the secondary metabolites [13,33]. Like other *Artemisia* species, *A. alba Turra* is a taxonomically problematic species due to its polymorphism [10]. In the present study, ethanol extracts from *A. alba Turra* were analyzed for the TPC and TFC. Previous studies found that *Artemisia* species are rich in flavonoids, caffeoylquinic acids, terpenoids, coumarins, acetylenes, and sterols. One of the most abundant bioactive components of *A. alba Turra* ethanol extract was phenolic compounds [34]. The phytochemical data on phytocompounds of *Artemisia* growing in Romania are limited. For the *A. alba Turra* harvested from the Alexandru Borza Botanical Garden, Cluj-Napoca, Romania, TPC was reduced, and TFC was consistent. The TPC of the present *A. alba Turra* ethanol extract (3.4 ± 0.21 mg GAE/g d.w. plant material) was significantly smaller than that of an *A. alba Turra* aqueous extract (83.59 ± 0.96 mg GAE/g d.w. plant material) of plants collected from Algeria [35], methanol extracts of *A. alba Turra* flowers (10.7 ± 0.4 mg GAE/g d.w. plant material) and *A. alba Turra* leaves (5.4 ± 0.3 mg GAE/g d.w. plant material) harvested from Bulgaria [36]. Compared to previous studies that also analyzed *A. alba* ethanol extract (27.65 mg GAE/g dry weight, 88 mg GAE/g dry weight), the TPC was smaller too [37].

Significant differences were also found in the case of TFC, respectively: the presented *A. alba Turra* ethanol extract had a significantly higher TFC (147.12 ± 10.09 mg QE/100 g d.w. plant material) than the *A. alba Turra* aqueous extract from Algeria (25.7 ± 0.95 QE/g d.w. plant material) [35]. The TFC of the methanol extract of *A. alba Turra* from Bulgaria expressed as mg catechin equivalents per 1 g of dry plant material was higher in the flower (2.9 ± 0.3 mg CE/g d.w. plant material) than in the leaves (1.8 ± 0.1 mg CE/g d.w. plant material) [36]. Taken together, due to the higher flavonoid content, these results suggest that *A. alba Turra* harvested from the Alexandru Borza Botanical Garden, Cluj-Napoca, Romania, can be a good flavonoid antioxidant source.

To provide new insights into the chemical polymorphism of *A. alba*, the chemical profile of the phenolic compounds from *A. alba Turra* ethanol extract was determined by HPLC-ESI MS analysis. Polyphenolic compounds are secondary metabolites that occur naturally in plants and have positive effects on human health. Of the more than 10,000 different polyphenols, most of them are mainly phenolic acids, flavonoids, and tannins. Phenolic acids are divided into two subgroups: hydroxybenzoic and hydroxycinnamic acids. Flavonoids are the largest group of polyphenols and are divided into six subclasses: anthocyanins, chalcones, flavanols or catechins, flavanones, flavones, and isoflavones [38]. Despite the differences in the analysis methods, the *A. alba Turra* ethanol extract of this study and a methanol extract of *A. alba* from another study had significant concentrations of chlorogenic acid, caffeoyl tartaric acid, 3,4-dicaffeoylquinic acid, 3,5-dicaffeoylquinic acid, and 4,5-dicaffeoylquinic acid [36]. In the literature, it was pointed out that the flavonoid profiles of *A. alba* from different geographical regions are different [33]. For example, *A. alba* from the Balkan Peninsula contained both flavones and flavonol types of compounds, and *A. alba* from the Mediterranean countries contained only flavonol types of compounds [13,39,40]. The data obtained in this study found that this Romanian *A. alba Turra* ethanol extract contained flavones and flavonols. These differences can be related to the specific chemotype of each extract, which is also influenced by the extraction solvent and technique.

Plant-derived compounds are an important source of medicines and have received significant attention in recent years due to their pharmacological properties [41]. It is well known that the antioxidant properties of plant extracts have been attributed to the high content of polyphenols [36,42]. Many plants from the *Asteraceae* family from the genus *Artemisia* are rich in such antioxidants [10,11,43]. In the present study, the in vitro antioxidant capacity of the *A. alba Turra* ethanol extract was evaluated using four assays: DPPH, FRAP, H_2_O_2_, and NO. The results of the present work show that *A. alba* samples possess a relatively moderate DPPH antiradical activity, although lower than that of Trolox. *A. alba Turra* flower and leaf methanol extract possessed higher DPPH radical scavenging activity, suggesting that a different solvent used in plant extract preparation could also impact the in vitro antioxidant assay results [37]. Another antioxidant test used in this study was the FRAP assay. The moderate antioxidant activity measured by the FRAP assay was smaller than that of Trolox, suggesting that more antioxidants from *A. alba Turra* ethanol extract are needed to donate a single electron or hydrogen atom for reduction [37]. H_2_O_2_ and NO radical scavenging activities of *A. alba Turra* ethanol extract were found to be moderate but significant. The presence of major phytocompounds may have been responsible for the in vitro antioxidant activity. The results of the present study’s in vitro antioxidants assays are consistent with prior research findings [44] and demonstrate that *A. alba Turra* ethanol extract possesses high antioxidant potential. Moreover, the good correlation between the in vitro antioxidant test results and the polyphenol concentration confirmed that these compounds are likely to contribute to the radical scavenging activity of this plant extract.

Ovarian cancer is the eighth most common cancer in women. Some plant extracts have anticancer effects, but the mechanisms have not been scientifically evaluated. The MTT test showed that *A. alba Turra* extract displayed in vitro antiproliferative activity against three human ovarian tumor cell populations: cisplatin resistance in A2780*cis* cells, the highly chemoresistant ovarian cancer cells OVCAR-3, and the moderately drug-sensitive tumors cells OAW-42. This diverse selection provides a comprehensive assessment of the extracts’ potential as anticancer agents across different ovarian cancer subtypes and resistance profiles. The A2780*cis* and OVCAR-3 cells were the most sensitive to the relative short-term exposure (72 h) to *A. alba Turra* extract. Two mechanisms were analyzed: the capacity of the extracts to modulate the MDR protein and the synthesis of NfkB-p65. The tumoral cell lines expressed a significant basal MDR protein level, which was downregulated by the *A. alba Turra* extract only at the higher tested dose. NfkB-p65 was detected in the untreated ovarian cell lysates. *A. alba Turra* extract strongly reduced NF-kB activation in A2780*cis* and OVCAR-3 cells at all concentrations, and OAW-42 cell lines were inhibited only by the highest extract concentration. These results denote that *A. alba Turra* extract has antiproliferative activity on ovarian cancer cells, but it acts through different mechanisms involved in cancer progression or treatment resistance, depending on the ovarian cancer cell type [21,22,23]. The cytotoxic effects of *A. alba Turra* extract were tested on colorectal carcinoma cells, and it was associated with apoptosis induction, cell cycle arrest, and the modulation of the PI3K/AKT/mTOR pathway [45,46].

Further in vivo experimental studies are being carried out to identify some mechanisms of anti-inflammatory and associated antioxidant potential. Many studies show that plant extract health benefits are correlated with polyphenols’ antioxidant and anti-inflammatory properties [38]. Analyzing ethnic uses of *A. herba-alba*, two reviews mentioned that phytochemicals can be useful against human diseases that involve oxidative stress and inflammation [47].

An aqueous *A. herba-alba* extract reduced oxidative stress by decreasing ROS generation [48,49]. In the present study, ROS production was evaluated with general parameters (TOS and OSI) and by measuring some important specific biomarkers for oxidative stress: MDA, AOPP, NO, 3NT, 8-OHdG. All *A. alba Turra* ethanol extract dilutions reduced oxidative stress by lowering TOS and OSI, but Trolox had a stronger inhibitory activity.

We recorded a significant increase in the lipid peroxides measured as serum MDA in INFL, and treatment with *A. alba Turra* ethanol caused just a small decrease. Similarly, there was no significant reduction in MDA in trolox-treated animals. Our findings align with those of other studies that confirm the antioxidant capacity of *A. herba-alba* against inflammation-induced oxidative stress by lowering MDA [42]. These results are important because MDA is a highly diffusible compound that may play a critical role in the development of the harmful effects of the lipid peroxides in the tissues and serum and may activate apoptosis through the intrinsic pathways that exist in all cells [50].

AOPPs, as plasma protein oxidation biomarkers, were increased by inflammation, and *A. alba Turra* ethanol extract significantly reduced it. Similar observations were found for an aqueous extract of *A. herba-alba* [42]. A positive consequence of AOPP reduction may be an improvement in antioxidant enzymes’ activity.

During inflammation, NO is produced from L-arginine by the inducible NO synthase (iNOS). If oxidative stress is associated, the rapid reaction between superoxide and NO leads to RNS formation and further tissue injury [51]. Peroxynitrite (ONOO^−^), an RNS, causes protein tyrosine nitration resulting in 3-nitrotyrosine (3NT) formation. NO and 3NT are sensitive biomarkers of RNS involvement in oxidative stress [51]. In the INFL group, NO and 3NT were increased, and *A. alba Turra* ethanol extract caused a dose-dependent reduction in NO and 3NT, the AAT 100% having the best inhibitory activity. Another study analyzing the effect of *A. herba-alba* aqueous extract found similar or better efficiency at higher doses [51]. *A. alba Turra* ethanol extract’s effect on NO and 3NT was considerably smaller than that of Trolox.

ROS can be responsible for DNA damage that may lead to mutations, epigenetic changes, and tumor development [48]. 8-OHdG increase is a marker of oxidative DNA damage in the INFL group. Only AAT 100% managed to significantly reduce 8-OHdG, and this effect was similar to that of Trolox. This result suggested that only the undiluted *A. alba Turra* ethanol extract may have efficient anticancer activity.

The antioxidant status was evaluated by measuring TAC. Inflammation reduced TAC, and *A. herba-alba* aqueous extract had no significant effect on it. Further, we determined SH as an important component contributing to TAC [52]. SH are known antioxidants that act through more mechanisms, such as components of the general thiol/disulfide redox buffer, metal chelators, radical quenchers, substrates for specific redox reactions, and specific reductants of individual protein disulfate bonds [53,54]. In the present study, *A. herba-alba* ethanol extract significantly increased serum SH, and the effect was better than that of Trolox.

Taken together, we concluded that *A. herba-alba* ethanol extract reduces oxidative stress mostly by lowering ROS and RNS and at a lower level by increasing the antioxidants. These findings are in accordance with the results obtained after treatment with other *Artemisia* plant extracts [55,56].

Inflammation is a complex network of interactions between parenchymal cells and resident immune cells, coupled with the recruitment of white blood cells. Once stimulated, these cells activate receptors that detect molecules derived from microorganisms that trigger immune responses (PAMPs) and molecules released by damaged or stressed cells (DAMPs) and further activate major innate immunity pathways, such as nuclear factor ĸB (NF-ĸB) and NLRP3 inflammasome [50]. In conditions of well-controlled inflammation, the process gradually subsides. Uncontrolled or prolonged inflammatory responses result in proinflammatory mediators that exacerbate tissue damage and disseminate into systemic circulation, potentially causing systemic inflammatory response syndrome, organ failure, and multiple organ dysfunction syndromes [57]. Therefore, the inhibition of NF-*κ*B expression and NLRP3 inflammasome activation are important targets for anti-inflammatory drug development.

The current anti-inflammatory treatment uses steroidal anti-inflammatory drugs (NSAIDs). The negative side effects of these drugs, such as kidney disorders and gastrointestinal ulcers, led to the search for new plant-derived anti-inflammatory compounds with no side effects [58].

In unstimulated cells, NF-κB subunits are restricted to the cytoplasm due to the inhibitory effects of the inhibitor of κB (IκB) family. At the cellular level, a complex mixture of stressful stimuli induces the disassociation of IκB and NF-κB heterodimers to move into the nucleus and bind to specific gene promoters to modulate the expression of pro- and anti-inflammatory proteins [59,60]. The p50 and p65 form the most common heterodimer in the NF-κB signaling pathway, and p65 is typically involved in the inflammatory response. Several species of *Artemisia* used in traditional medicine proved to have anti-inflammatory activity [61]. Artemisinin, other sesquiterpene lactones and quercetin from *Artemisia* plants are NF-*κ*B inhibitors [62,63]. *A. herba-alba* decocts showed a moderate anti-inflammatory effect, and this effect was attributed to flavonoids present in the aqueous extract [64]. Notably, the present study reveals that *A. alba Turra* ethanol extract exhibits anti-inflammatory activity by reducing NfkB-p65, and this may be linked with the high flavonoid content. This mechanism is important because NF-*κ*B controls the response of many inflammatory mediators, such as cytokines, chemokines, and adhesion molecules.

Inflammasomes are cytosolic supramolecular complexes of the innate immune system. The NLRP3 inflammasome is one of the most studied inflammasomes and functions as a detector for cellular stress and cell membrane damage. It contains an NLRP3 sensor, ASC adaptor, and caspase-1 protease. NLRP3 activation requires two steps: a priming step and an activation step. In the priming step, after the activation of NF-κB, the expression of NLRP3 is transcriptionally upregulated. In the second step, NLRP3 is activated [65]. Upon activation, NLRP3 assembles to form the inflammasome complex and activate caspase-1. The activated caspase-1 leads to the proteolytic activation of IL-1β, IL-18, gasdermin D, and pyroptosis, an inflammatory cell death mechanism [66]. NLRP3 inflammasome has been implicated in the pathogenesis of many diseases [67]. In the present study, *A. alba Turra* ethanol extract’s inhibitory activity upon the NLRP3 inflammasome was indicated by significant reduction in IL-1b, IL-18, caspase-1, and GSDMD. This may be the consequence of the polyphenol content of the extract, considering that more than 8000 polyphenols have been identified as potential NLRP3 inflammasome inhibitors [68,69]. Regarding IL-18 *A. alba Turra* ethanol extract, its inhibitory activity was better than that of diclofenac, a classical nonsteroidal anti-inflammatory drug.

Considering that OS and inflammation are inseparably linked, meaning that pathological inflammation triggers OS, and OS triggers inflammation [34], the antioxidant and anti-inflammatory effects of *A. alba Turra* ethanol extract may explain the efficacy of this plant product. At the same time, these effects may recommend *A. alba Turra* ethanol extract as a complementary therapy targeting tumor-induced inflammation in the TME.

There are two limitations for plant-derived medications: clinical trials on these treatments are rare, and some plant medications have undetermined toxicity [14,58]. In the present study, the chemotype and some pharmacological activities were evaluated upon considering renal and liver toxicity.

The inflammatory cytokines are distributed in the whole body. The kidney receives 25% of the entire blood volume, without having antioxidant and anti-inflammatory defense mechanisms, such as hepatic tissue. Therefore, OS and inflammatory mediators can influence the intrarenal microcirculation and can induce renal injury and even renal failure [70]. It was reported that *A. herba-alba* aqueous extract reduced ROS production and increased the antioxidant defense in an experimental diabetes mellitus [35]. In our study, the renal damage markers urea and creatinine were significantly increased by inflammation. Conversely, we noted an important reduction in serum urea and creatinine after *A. alba Turra* ethanol extract administration. The renal protecting activity was correlated with improvements in the OS and inflammatory tests, indicating that renal dysfunction was a consequence of inflammation and inflammation-induced OS.

In the liver injury tests, AST and ALT showed no significant increase in INFL animals and all *A. alba Turra* ethanol extract-treated groups.

The toxicity results indicated the safety of the *A. alba Turra* ethanol extract used in the present experimental conditions.

A general major concern of polyphenol use in therapy and a limit of the present study is the low bioavailability of these compounds due to their poor absorption, rapid metabolism, and systemic elimination. Therefore, more research is needed to explore the effects of *A. alba Turra* ethanol extract incorporated in nanoparticles or liposome as a better future therapeutic approach.

## 5. Conclusions

The chemotype analysis of the ethanol extract of *A. alba Turra* harvested from the Alexandru Borza Botanical Garden, Cluj-Napoca, Romania, showed that it has a lower phenolic acid and higher flavonoid content compared to *A. alba* extracts from other geographical regions. This *A. alba Turra* ethanol extract had antiproliferative activity against some ovarian cancer cells by reducing MDR protein and NF-*κ*B expression. The *A. alba Turra* ethanol extract reduced OS mostly by lowering ROS production and at a lower level by increasing the antioxidants. The inflammatory biomarkers indicated that in rat turpentine oil-induced inflammation, *A. alba Turra* ethanol extract anti-inflammatory activity was associated with NF-*κ*B and NLRP3 inflammasome inhibition.

Considering the efficacy of this Romanian *A. alba Turra* ethanol extract, its antiproliferative, antioxidant, and anti-inflammatory activities, and the safety of this plant medicine, it may be recommended as an adjuvant therapy in inflammatory diseases and cancer. Further studies are necessary to confirm these findings.

## Figures and Tables

**Table 1:** *Artemisia alba Turra* ethanol extract phenolic compound tentative identification and quantification (μg/g) by HPLC-MS.

Peak No.	R_t_ (min)	UV λ_max_ (nm)	[M + H]^+^ (*m*/*z*)	Compound	Subclass	*A. alba Turra*
1	3.22	275	155	3,5-Dihydroxybenzoic acid	Hydroxybenzoic acid ^1^	74.138 ± 2.12
2	11.04	323	355, 163	3-Caffeoylquinic acid (neochlorogenic acid)	Hydroxycinnamic acid ^2^	290.674 ± 15.66
3	12.57	323	355, 163	5-Caffeoylquinic acid (chlorogenic acid)	Hydroxycinnamic acid ^2^	4843.820 ± 82.94
4	12.95	323	355, 163	4-Caffeoylquinic acid (criptochlorogenic acid)	Hydroxycinnamic acid ^2^	161.934 ± 8.73
5	13.21	323	343, 163	Caffeoyl acid-glucoside	Hydroxycinnamic acid ^2^	550.971 ± 22.70
6	13.72	324	195	Iso-Ferulic acid	Hydroxycinnamic acid ^2^	160.712 ± 6.78
7	14.24	325	313	Caffeoyl tartaric acid	Hydroxycinnamic acid ^2^	1033.149 ± 46.69
8	14.59	330, 270	565, 271	Apigenin-arabinosyl-glucoside	Flavone ^3^	106.114 ± 3.04
9	15.02	324	369, 195	3-Feruloylquinic acid	Hydroxycinnamic acid ^2^	442.686 ± 14.24
10	15.47	324	369, 195	4-Feruloylquinic acid	Hydroxycinnamic acid ^2^	285.520 ± 10.31
11	15.88	324	369, 195	5-Feruloylquinic acid	Hydroxycinnamic acid ^2^	187.065 ± 3.03
12	16.03	255, 360	611, 303	Quercetin-rutinoside (rutin)	Flavonol ^4^	681.141 ± 14.11
13	16.66	330, 270	565, 271	Apigenin-glucosyl-arabinoside	Flavone ^3^	125.737 ± 4.50
14	17.13	240, 350	625, 317	Isorhamnetin-rutinoside	Flavonol ^4^	1348.303 ± 80.81
15	17.52	323	517, 163	3,4-Dicaffeoylquinic acid	Hydroxycinnamic acid ^2^	1524.679 ± 98.54
16	17.86	323	517, 163	3,5-Dicaffeoylquinic acid	Hydroxycinnamic acid ^2^	2654.119 ± 97.89
17	18.11	323	517, 163	Quinic acid derivative	Hydroxycinnamic acid ^2^	972.472 ± 57.03
18	18.35	323	517, 163	4,5-Dicaffeoylquinic acid	Hydroxycinnamic acid ^2^	1369.054 ± 87.50
19	18.73	324	531, 163	3-Feruloyl-4-caffeoylquinic acid	Hydroxycinnamic acid ^2^	312.830 ± 15.64
20	19.42	324	531, 163	4-Feruloyl-5-caffeoylquinic acid	Hydroxycinnamic acid ^2^	153.220 ± 3.98
21	19.84	324	545, 163	3,4-Diferuloylquinic acid	Hydroxycinnamic acid ^2^	308.952 ± 13.67
22	20.44	324	545, 163	3,5-Diferuloylquinic acid	Hydroxycinnamic acid ^2^	171.285 ± 4.62
23	21.32	324	531, 163	5-Caffeoyl-4-feruloyl-quinic acid	Hydroxycinnamic acid ^2^	196.257 ± 5.73
24	22.91	323	679, 163	3,4,5-Tricaffeoylquinic acid	Hydroxycinnamic acid ^2^	193.654 ± 2.34
25	23.68	330, 270	361	3,5-Dihydroxy-6,7,4′-trimethoxyflavone	Flavone ^3^	93.217 ± 5.02
26	24.39	330, 270	375	3,5-Dihydroxy-6,7,3′,4′-tetramethoxyflavone	Flavone ^3^	27.307 ± 2.77

^1^ Hydroxybenzoic acids were calculated as gallic acid equivalents (R^2^ = 0.9978; LOD = 0.35 μg/mL, LOQ = 1.05 μg/mL. ^2^ Hydroxycinnamic acids were quantified as chlorogenic acid equivalents (R^2^ = 0.9937), LOD = 0.41 μg/mL, LOQ = 1.64 μg/mL. ^3^ Flavones were quantified as luteolin equivalents (R^2^ = 0.9972), LOD = 0.26 μg/mL, LOQ = 0.95 μg/mL. ^4^ Flavonols were quantified as rutin equivalents (R^2^ = 0.9981), LOD = 0.21 μg/mL, LOQ = 0.84 μg/mL.

**Table 2:** The in vitro antioxidant activity of the *Artemisia alba Turra* ethanol extract.

	DPPH μg TE/mL	FRAP mg TE/mL	H_2_O_2_ Scavenging Activity μg TE/mL	NO Scavenging Activity μg QE/mL
*A. alba Turra* (1 g/1.2 mL)	42.66 ± 0.53	54.91 ± 0.56	38.48 ± 0.40	66.55 ± 1.28
TROLOX (mg)	11.61 ± 0.14	15.28 ± 1.15	12.04 ± 0.12	
Quercitin (mg)				20.05 ± 0.18

Note: Values are expressed as mean ± SD (n = 3). *A. alba Turra* extract 1 g/1.2 mL^−1^ g of fresh plant in 1.2 mL of final extract. DPPH—DPPH radical scavenging activity; FRAP—ferric reducing antioxidant power; H_2_O_2_—hydrogen peroxide scavenging activity; NO—nitric oxide radical scavenging activity; TE—TROLOX equivalent; QE—quercitin equivalent.

**Table 3:** In vivo antioxidant activity biomarkers of the study groups.

Groups	TOS (µmol H_2_O_2_E/L)	TAC (mmol TE/L)	OSI	AOPP (µmol/L)	MDA (nmol/L)	NO (µmol/L)	3NT (ng/mL)	8-OhdG (ng/mL)	SH (µmol/L)
CONTROL	14.72 ± 2.34	1.08 ± 0.00	15.52 ± 2.16	26.93 ± 1.70	2.54 ± 0.16	25.26 ± 3.41	22.14 ± 2.35	24.16 ± 1.89	340.24 ± 30.14
INFL	50.00 ± 4.93 ^a^	1.17 ± 0.08 ^a^	43.09 ± 4.00 ^a^	68.28 ± 6.19 ^a^	4.08 ± 0.35 ^a^	37.00 ± 6.25 ^a^	70.24 ± 5.32 ^a^	87.64 ± 11.72 ^a^	249.24 ± 18.61 ^a^
AAT 100%	25.75 ± 1.46 ^b,c^	1.09 ± 0.00	23.67 ± 3.70 ^b,c^	37.51 ± 3.75 ^b^	2.62 ± 0.20 ^b^	41.60 ± 7.94 ^b,d^	45.10 ± 2.58 ^b^	44.49 ± 3.09 ^b^	389.50 ± 17.98 ^b,c,d^
AAT 50%	27.68 ± 3.75 ^b,c^	1.09 ± 0.00	25.48 ± 2.14 ^b,c^	35.81 ± 2.08 ^b^	2.78 ± 0.19 ^b^	44.00 ± 9.86 ^b,d^	59.44 ± 6.96 ^c,d^	68.34 ± 6.83 ^b,c,d^	417.80 ± 34.70 ^b,c,d^
AAT 25%	19.09 ± 2.76 ^b^	1.08 ± 0.00	17.61 ± 1.16 ^b^	29.65 ± 1.76 ^b^	2.46 ± 0.22 ^b^	51.62 ± 4.83 ^d^	53.99 ± 4.10 ^c,d^	67.68 ± 6.24 ^b,c,d^	309.40 ± 29.93 ^b^
DICLO	20.24 ± 2.11 ^b^	1.09 ± 0.00 ^b^	15.08 ± 1.66 ^b^	25.85 ± 1.63 ^b^	2.89 ± 0.12 ^b^	25.41 ± 3.26 ^b^	30.22 ± 2.34 ^b^	48.12 ± 5.04 ^b^	260.17 ± 27.44
TX	18.16 ± 1.17 ^b^	1.09 ± 0.00 ^b^	15.17 ± 1.92 ^b^	27.62 ± 2.60 ^b^	2.72 ± 0.24 ^b^	38.54 ± 4.23 ^b^	20.48 ± 2.72 ^b^	40.06 ± 4.91 ^b^	280.86 ± 22.45

Note: Values are expressed as mean ± SD (n = 9). Statistical significance: vs. CONTROL: ^a^ *p* <  0.05; vs. INFL: ^b^ *p* <  0.05; vs. TX: ^c^ *p* <  0.05; vs. DICLO: ^d^ *p* <  0.05; INFL—inflammation group; AAT 100%—*Artemisia alba Turra* undiluted extract; AAT 50%—*Artemisia alba Turra* 1:2 diluted extract; AAT 25%—*Artemisia alba Turra* 1:4 diluted extract; TX—Trolox; DICLO—diclofenac; TOS—total oxidative status; TAC—total antioxidant capacity; OSI—oxidative stress index; AOPP—advanced oxidation protein product; MDA—malondialdehyde; NO—nitrites and nitrates; 3NT—3-nitrotyrosine; 8-OHdG—8-hydroxydeoxyguanosine; SH—total thiols.

**Table 4:** In vivo anti-inflammatory activity biomarkers of the study groups.

Groups	NfkB-p65 (ng/mL)	IL-1b (pg/mL)	IL-18 (pg/mL)	Caspase—1 (pg/mL)	GSDMD (ng/mL)
CONTROL	138.26 ± 10.09	22.13 ± 1.87	20.05 ± 1.09	12.52 ± 2.00	4.77 ± 0.35
INFL	329.57 ± 20.13 ^a^	60.16 ± 4.22 ^a^	60.29 ± 8.41 ^a^	130.74 ± 10.25 ^a^	10.13 ± 0.86 ^a^
AAT 100%	138.51 ± 15.33 ^b^	27.29 ± 1.57 ^b^	22.60 ± 2.70 ^b,c^	49.29 ± 6.52 ^b^	5.44 ± 0.34 ^b^
AAT 50%	187.23 ± 18.76 ^b^	30.42 ± 2.07 ^b^	25.20 ± 1.15 ^b,c^	55.22 ± 4.82 ^b^	6.03 ± 0.51 ^b^
AAT 25%	754.38 ± 14.18 ^b^	31.46 ± 4.48 ^b^	28.54 ± 3.52 ^b,c^	51.13 ± 2.22 ^b^	7.09 ± 0.84 ^b^
DICLO	135.22 ± 10.41 ^b^	25.81 ± 2.44 ^b^	48.87 ± 2.76 ^b,c^	42.07 ± 4.83 ^b^	5.16 ± 0.61 ^b^
TX	150.15 ± 10.28 ^b^	30.42 ± 4.06 ^b^	28.46 ± 2.72 ^b^	50.42 ± 4.21 ^b^	5.53 ± 0.54 ^b^

Note: Values are expressed as mean ± SD (n = 9). Statistical significance: vs. CONTROL: ^a^ *p* <  0.05; vs. INFL: ^b^ *p* <  0.05; vs. DICLO: ^c^ *p* <  0.05; INFL—inflammation group; AAT 100%—*Artemisia alba Turra* undiluted extract; AAT 50%—*Artemisia alba Turra* 1:2 diluted extract; AAT 25%—*Artemisia alba Turra* 1:4 diluted extract; TX—Trolox; DICLO—diclofenac; NfkB-p65—Nuclear factor-κB; IL-1b—Interleukine 1-b; IL-18—Interleukine 18; GSDMD—Gasdermine D.

**Table 5:** Liver and renal injury biomarkers of the study groups.

Groups	ALT (U/L)	AST (U/L)	Creatinine (mg/dL)	Urea (mg/dL)
CONTROL	43.12 ± 2.23	58.25 ± 4.18	0.72 ± 0.01	32.42 ± 3.02
INFL	49.87 ± 3.71	49.58 ± 4.09	1.05 ± 0.18 ^a^	57.32 ± 6.10 ^a^
AAT 100%	49.33 ± 4.95	54.95 ± 7.01	0.74 ± 0.11 ^b^	47.06 ± 6.86 ^b^
AAT 50%	38.12 ± 2.62	42.68 ± 6.09	0.88 ± 0.09 ^b^	39.56 ± 4.05 ^b^
AAT 25%	43.82 ± 2.33	39.56 ± 5.49	0.84 ± 0.09 ^b^	44.98 ± 3.19 ^b^
DICLO	35.24 ± 2.26	34.36 ± 3.05	0.72 ± 0.01 ^b^	42.06 ± 3.14 ^b^
TX	35.46 ± 2.09	32.53 ± 2.72	0.74 ± 0.07 ^b^	41.29 ± 3.11 ^b^

Note: Values are expressed as mean ± SD (n = 9). Statistical significance: vs. CONTROL: ^a^ *p* < 0.05; vs. INFL: ^b^ *p* < 0.05; INFL—inflammation group; AAT 100%—Artemisia alba Turra undiluted extract; AAT 50%—Artemisia alba Turra 1:2 diluted extract; AAT 25%—Artemisia alba Turra 1:4 diluted extract; TX—Trolox; DICLO—diclofenac; ALT—alaninaminotransferase; AST—aspartataminotransferase.

## Data Availability

The original contributions presented in the study are included in the article/Supplementary Materials, further inquiries can be directed to the corresponding author.

## Supplementary Materials

The following supporting information can be downloaded at: https://www.mdpi.com/article/10.3390/foods14081389/s1, Figure S1: The modulation of intracellular multidrug resistance-related MDR1 concentration expressed as percent from the untreated control cells relative fluorescence intensity in ovarian tumour cell lines A2780cis, OVCAR-3 and OAW-42 treated for 72 h with Artemisia alba Turra extracts at four different concentrations (3.13; 6.25; 12.5 and 25.00 μg/mL); Figure S2: Modulation of intracellular NF-κB expressed as the optical density (OD) in tumour cell lines A2780cis, OVCAR-3, OAW-42, and in the normal HaCaT cell line treated for 72 hours with Artemisia alba Turra extracts related to the untreated cells from each cell line.
