# Supplementary material for: The Phytochemical and Functional Characterization of the Aerial Parts of Artemisa alba Turra (Asteraceae) Grown in Romania
Source: Foods. 2025 Apr 17;14(8):1389. doi: 10.3390/foods14081389 (PMC12027346; doi:10.3390/foods14081389)
Supplement: Supplementary file 1 [file foods-14-01389-s001.zip › foods-3544868-supplementary.pdf]

Supplementary Files

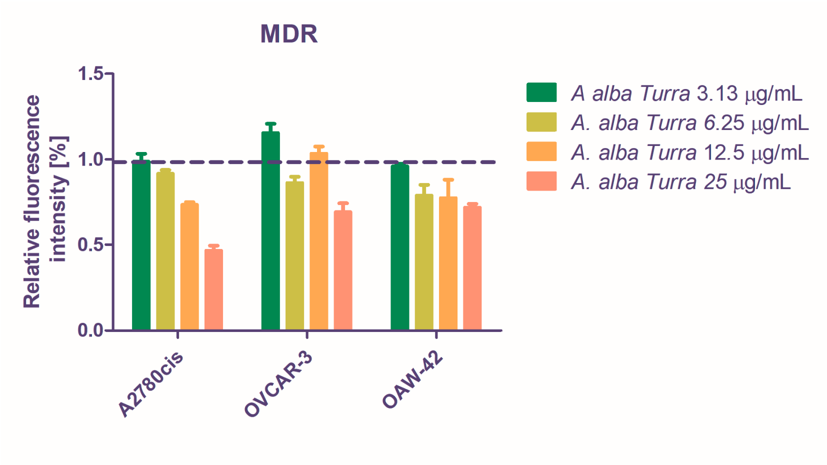

Figure S1. The modulation of intracellular multidrug resistance-related MDR1 concentration expressed as percent from the untreated control cells relative fluorescence intensity in ovarian tumour cell lines A2780cis, OVCAR-3 and OAW-42 treated for 72 hours with A*rtemisia alba Turra* extracts at four different concentrations (3.13; 6.25; 12.5 and 25.00 μg/mL).

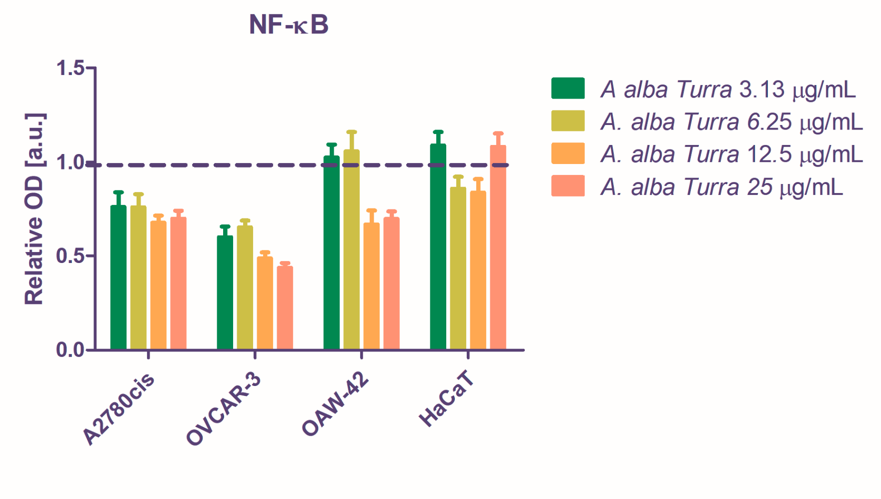

Figure S2. Modulation of intracellular NF-κB expressed as the optical density (OD) in tumour cell lines A2780cis, OVCAR-3, OAW-42, and in the normal HaCaT cell line treated for 72 hours with *Artemisia alba Turra* extracts related to the untreated cells from each cell line
